# *In Vitro* Selection of Mutant HDM2 Resistant to Nutlin Inhibition

**DOI:** 10.1371/journal.pone.0062564

**Published:** 2013-04-30

**Authors:** Siau Jia Wei, Thomas Joseph, Adelene Y. L. Sim, Larisa Yurlova, Kourosh Zolghadr, David Lane, Chandra Verma, Farid Ghadessy

**Affiliations:** 1 p53Lab, Agency for Science, Technology and Research, Singapore, Singapore; 2 Bioinformatics Institute, Agency for Science, Technology and Research, Singapore, Singpore; 3 ChromoTek GmbH, Planegg-Martinsried, Germany; 4 School of Biological Sciences, Nanyang Technological University, Singapore, Singapore; 5 Department of Biological Sciences, National University of Singapore, Singapore, Singapore; Rush University Medical Center, United States of America

## Abstract

HDM2 binds to the p53 tumour suppressor and targets it for proteosomal degradation. Presently in clinical trials, the small molecule Nutlin-3A competitively binds to HDM2 and abrogates its repressive function. Using a novel *in vitro* selection methodology, we simulated the emergence of resistance by evolving HDM2 mutants capable of binding p53 in the presence of Nutlin concentrations that inhibit the wild-type HDM2-p53 interaction. The *in vitro* phenotypes were recapitulated in *ex vivo* assays measuring both p53 transactivation function and the direct p53-HDM2 interaction in the presence of Nutlin. Mutations conferring drug resistance were not confined to the N-terminal p53/Nutlin–binding domain, and were additionally seen in the acidic, zinc finger and RING domains. Mechanistic insights gleaned from this broad spectrum of mutations will aid in future drug design and further our understanding of the complex p53-HDM2 interaction.

## Introduction

The p53 tumour suppressor functions as a master regulator of cell fate [1], [2] and is commonly mutated in cancer [3], [4]. Its pro-apoptotic activity is negatively regulated by HDM2, the ubiquitin-ligase that binds to p53 and targets it for proteosomal degradation [5]–[8]. Approximately 50% of cancers harbor wild-type p53, and elevation of p53 levels in these cancers by targeted disruption of the HDM2-p53 complex represents an attractive therapeutic modality [9]. Numerous agents including peptides, stapled peptides, mini-proteins, and small molecules have been described which bind to the p53-binding pocket in the N-terminal domain of HDM2 [10]–[12]. Occlusion of the p53 binding pocket results in rapid elevation of p53 levels, with the attendant downstream expression of proteins eliciting cell-cycle arrest and/or cell death.

The small molecule Nutlin-3A (hereafter referred to as Nutlin) binds to the p53-binding pocket in the N-terminal domain of HDM2 by mimicking core interactions of residues in the p53 transactivation domain that interact with the pocket [9]. Both Nutlin and related imidazoline compounds are presently in advanced preclinical development and clinical trials for the treatment of retinoblastoma, blood malignancies and liposarcoma with wild-type p53 status [13]–[15].

Recent *ex vivo* studies have indicated that prolonged exposure of cells to sub-lethal doses of Nutlin can result in acquired resistance through de novo inactivating mutations of p53 or endoreduplication [16], [17]. Whilst these studies did not investigate HDM2 status, mutant HDM2 has been previously identified in tumour samples [18], [19]. Furthermore, HDM2 gene amplification and over-production in cancer [20], [21], and correlation with poor response to therapy [22], suggests that HDM2 mutations could render cells recalcitrant to Nutlin therapy. To investigate this possibility in a targeted manner, it would therefore be desirable to interrogate large numbers of mutated HDM2 variants for a Nutlin-resistance phenotype, wherein the interaction with p53 is not attenuated by the drug [23]. We have previously described the use of *in vitro* compartmentalization (IVC), a completely cell-free method utilizing the discrete aqueous compartments of a water-in-oil emulsion to select for p53 variants with altered DNA binding specificities [24]. In the present study, we have adapted the selection protocol to enable selection of HDM2 variants able to bind p53 in the presence of Nulin from an exceptionally large mutant repertoire. Analysis of the selectants identified mutations not only in the N-terminal domain that binds p53, but also in the acidic, zinc finger and RING domains which gave the Nutlin-resistant phenotype. Furthermore, this phenotype was recapitulated in *ex vivo* assays measuring p53 transactivation function and the formation of p53-HDM2 complexes in the presence of Nutlin.

## Materials and Methods

### Materials

Unless otherwise specified, all oligonucleotides used in this work were from 1^st^ Base (Singapore), restriction enzymes from NEB and chemical reagents from Sigma. Nutlin-3A was from Calbiochem.

### Primers

petF3conA-Rlink: 5′- GTGACTCAGCGGACATGCCCGGACATGCCCCAGGTGCGGTTGCTGGCGCCTAT -3′

petF4conA-Flink: 5- GCTGAGTCACGGGCATGTCCGGGCATGTCCGATGCGTCCGGCGTAGAGGATCG -3′

petF2∶5′- CATCGGTGATGTCGGCGAT -3′

petR: 5′- CGGATATAGTTCCTCCTTTCAGCA -3′

Hdm2-Nde1∶5′- CACAACATATGTGCAATACCAACATGTCTGTACC -3′

Hdm2-HA-BamH1∶5′- GCTCTGGATCCTTAAGCGTAATCTGGAACATCGTATGGGTAGGGGAAATAAGTTA -3′

INF-Hdm2-cmvF: 5′- CGAACCTAAAAACAAATGTGCAATACCAACATGTCTGTAC -3′

INF-HA-cmvRcor: 5′- TTATAGACAGGTCAACTAAGCGTAATCTGGAAC -3′

mdm2-T16A-QC1∶5′- GATGGTGCTGTAACCGCCTCACAGATTCCAG -3′

mdm2-T16A-QC2∶5′- CTGGAATCTGTGAGGCGGTTACAGCACCATC -3′

mdm2-P20L-QC1∶5′- CCACCTCACAGATTCTAGCTTCGGAACAAGA -3′

mdm2-P20L-QC2∶5′- TCTTGTTCCGAAGCTAGAATCTGTGAGGTGG -3′

mdm2-Q24R-QC1∶5′- TTCCAGCTTCGGAACGAGAGACCCTGGTTAG -3′

mdm2-Q24R-QC2∶5′- CTAACCAGGGTCTCTCGTTCCGAAGCTGGAA -3′

HDMM62A-1∶5′-CTTGGCCAGTATATTGCGACTAAACGATTATATG-3′

HDMM62A-2∶5′-CATATAATCGTTTAGTCGCAATATACTGGCCAAG-3′

mdm2-M62V-QC1∶5′- CTTGGCCAGTATATTGTGACTAAACGATTAT -3′

mdm2-M62V-QC2∶5′- ATAATCGTTTAGTCACAATATACTGGCCAAG -3′

mdm2-L82P-QC1∶5′- GTTCAAATGATCTTCCAGGAGATTTGTTTGG -3′

mdm2-L82P-QC2∶5′- CCAAACAAATCTCCTGGAAGATCATTTGAAC -3′

mdm2-V280A-QC1∶5′- TATATCAAGTTACTGCGTATCAGGCAGGGGA -3′

mdm2-V280A-QC2∶5′- TCCCCTGCCTGATACGCAGTAACTTGATATA -3′

mdm2-G443D-QC1∶5′- GTGTGATTTGTCAAGATCGACCTAAAAATGG -3′

mdm2-G443D-QC2∶5′- CCATTTTTAGGTCGATCTTGACAAATCACAC -3′

2CONART-F: 5′- GGCATGTCCGCTGAGTC -3′

WpetR1∶5′- TAATTTCGCGGGATCGAGATCT -3′

### Vector and HDM2 Library Construction

Inverse PCR was carried out on vector PET22b with primers petF3conA-Rlink and petF4conA-Flink and the PCR products were ligated intramolecularly to construct 2ConA-PET22b. The same inverse PCR was carried out on HDM2-PET22b to construct 2ConA-HDM2-PET22b. The HDM2-pet22b construct additionally encodes a C-terminal HA tag.

Error-prone PCR [25] was carried out on HDM2-PET22b using primers petF2 and petR and mutant genes re-amplified with Hdm2-Nde1 and Hdm2-HA-BamH1. The library was ligated into 2ConA-PET22b via Nde1/BamH1 sites and re-amplified with petF2 and petR to make library amplicons with T7 promoter and ribosome binding site required for *in vitro* transcription-translation (IVT), as well as the 2ConA RE site located before the T7 promoter site. Both 2ConA-HDM2-PET22b, HDM2-PET22b and p53-PET22b were also amplified with petF2 and petR for IVT of wild-type HDM2 and p53.

Nutlin-resistant parental clones obtained from the selection were amplified with petF2 and petR to create amplicons for secondary assays. Three parental clones (5–3, 5–9 and 5–14) were also amplified with INF-Hdm2-cmvF and INF-HA-cmvRcor for cloning by infusion (Clontech) into the pCMV expression vector.

Single mutant HDM2 clones were generated by Quickchange mutagenesis (Stratagene) of parental 2ConA-HDM2-PET22b using appropriate primers pairs. The same primers were used to introduce mutations into the parental pCMV-HDM2 mammalian expression construct.

### *In vitro* Selection of HDM2 Variants Resistant to Nutlin

IVT reactions consisting of 0.5 µM ZnCl_2_, 1 mM Nutlin, 8 ng p53 (1.6 ng in rounds 2/3, 0.8 ng in rounds 4/5), 5 ng library amplicons (1.0 ng in rounds 2/3, 0.5 ng in rounds 4/5) in a total volume of 50 µL PURExpress® *in vitro* protein synthesis solution (New England Biolabs) were assembled on ice and emulsified as previously described [24]. After incubation at 37°C, the reactions were centrifuged at 8000rpm for 10 mins to separate the aqueous and oil phase. The oil phase was removed and 50uL TNTB buffer (0.1 M Tris pH 7.4, 0.15 M NaCl, 0.05% Tween-20, 0.5% BSA) was added to the pellet of aqueous phase compartments. The compartments were disrupted by six rounds of hexane extraction and the aqueous phase incubated with anti-HA antibody-coated protein G beads (Invitrogen) at 4°C with rotation. The beads were washed thrice with PBST-0.1%BSA, and thrice with PBST. The beads were resuspended in 20 µl water and the protein-protein-DNA complexes eluted by incubation at 95°C for 5 mins. The eluates were amplified with Hdm2-Nde1 and Hdm2-HA-BamH1 and products cloned back into 2ConA-PET22b via Nde1/BamH1 sites and re-amplified with petF2 and petR for the next round of selection.

### Secondary Co-immunoprecipitation Assay and Western Blot Analysis

Protein G beads were incubated with anti-HA (1 µg per 10 µL beads) for 1 hour in PBST-3%BSA and subsequently washed twice in PBST-0.1%BSA. IVT-expressed protein was incubated with the beads on a rotator for 30 mins. Nutlin was added at required concentrations and incubation carried out for 30 mins. IVT-containing secondary protein was added to the mixture and incubation allowed for 1 hour. Beads were finally washed thrice in PBST-0.1%BSA and thrice with PBS, and bound proteins eluted by resuspension in 20 µL SDS-PAGE loading buffer and incubation at 95°C for 5 minutes. Where required, blank IVT extract (no template DNA added) was used as control. The eluates were subjected to electrophoresis, transferred to nitrocellulose membranes and probed for p53 with horseradish peroxidise conjugated DO1 antibody (Santa Cruz) or for HDM2 with anti-HA antibody followed by rabbit anti-mouse (Dakocytomation). For densitometric quantification, image acquisition was carried out using the LAS-4000 image reader (FujiFilm). Analysis was carried out with the Multiguage software package (FujiFilm).

### Proof-of-principle DNA Binding Assay and Real-time PCR

Protein G beads were incubated with anti-HA (1 µg per 5 µL beads) for 1 hour in PBST-3%BSA and subsequently washed twice in PBST-0.1%BSA. IVT-expressed HDM2 (with either HDM2 or HDM2 2ConA as template DNA) was incubated with the beads on a rotator for 30 mins. Nutlin was added at required concentrations and incubation carried out for 1 hour. IVT-expressed p53 was added to the mixture and incubation allowed for 1 hour. Beads were finally washed as above, and bound DNA eluted by resuspension in 20 µL nuclease-free water and incubation at 95°C for 5 minutes. Real-time PCR quantifications of the eluates were performed using 250 nM each of primers 2CONART-F and WpetR1 using iQTM SYBR® Green Supermix (Bio-Rad Laboratories) and quantified via CFX96 Real-Time System (Bio-Rad Laboratories). Data was interpreted as fold differences (calculated based on cycle threshold differences) over non-specific DNA binding control (HDM2 DNA).

### Cell Culture and Reporter Assay

Mouse embryonic fibroblast p53/Mdm2 double-knockout (DKO) cells (a kind gift from Guillermina Lozano) [26] and H1299 p53^−/−^ cells [27] were maintained in Dulbecco's modified Eagle's medium (DMEM) with 10% (v/v) foetal calf serum (FCS) and 1% (v/v) penicillin/streptomycin. The cells were seeded at 1.0×10^5^ cells/well in 6-well plates, 24 hours prior to transfection. Cells were co-transfected with parental or individual Nutlin-resistant HDM2 plasmid, p53-pcDNA plasmid, LacZ reporter plasmid and luciferase transfection efficiency plasmid using TurboFect transfection reagent (Thermo Scientific) according to the manufacturer’s instructions. Nutlin was added to selected wells at required concentrations 4.5 hours post-transfection. In all cases, the total amount of plasmid DNA transfected per well was equilibrated by addition of the parental vector pcDNA3.1a (+).

### β-Galactosidase Assay and Western Blot Analysis

DKO cells were harvested 24hours after transfection and β-galactosidase activities were assessed using the Dual-light System (Applied Biosystems) according to the manufacturer's protocol. The β-galactosidase activity was normalized with luciferase activity for each sample. To check for expression levels of relevant proteins via western blot, 2.5 µg of the cell lysates were probed for p53 with horseradish peroxidise conjugated DO1 antibody, for HDM2 and actin with anti-HA antibody and AC15 antibody respectively followed by rabbit anti-mouse.

### F2H Co-Localization Assay

Transgenic BHK cells [28] were co-transfected with plasmids encoding the bait p53 (amino acids 1–81) fusion protein and different prey HDM2 (amino acids 7–134) fusion proteins overnight in 96 multiwell plates (µClear Greiner Bio-One, Germany) using the Lipofectamine 2000 (Life Technologies) reverse transfection protocol according to manufacturer’s instructions with 0.2 µg DNA and 0.4 µl Lipofectamine 2000 per well. Cells were incubated with a dilution series of 50 µM, 10 µM, 2 µM, 1 µM, 0.5 µM, 0.25 µM and 0.13 µM Nutlin for 1 hour at 37°C, 5% CO_2_.

Interaction (%) was determined as the ratio of cells showing co-localization of fluorescent signals at the nuclear spot to the total number of evaluated cells. For automated image acquisition an INCell Analyzer 1000 with a 20X objective (GE Healthcare) was used. Automated image segmentation and analysis was performed with the corresponding INCell Workstation 3.6 software. At least 100 co-transfected cells were analyzed per well. Titrations were carried out independently three to five times.

### Statisitical Analysis

Statistical analysis (2-tailed independent student t-test) was carried out using the Excel software package. In cell-based reporter assays significance is denoted relative to corresponding data point(s) on graph for wild-type HDM2-p53 interaction. For the F2H assay, significance is denoted relative to the no-Nutlin treatment data point for each mutant tested.

### Molecular Dynamics Simulations

Interactions between the N terminal domain of HDM2 and the N terminal domain of p53 or Nutlin:

To model the interactions of the N terminal domain of HDM2 with p53 and Nutlin, the crystal structures of the HDM2-p53 complex [29] (PDB code 1YCR, resolved at 2.6 Å) and the HDM2-Nutlin complex [9] (PDB code 1RV1, resolved at 2.3 Å) were used. The N terminus of HDM2 was extended from residue 25 (as in 1YCR) by grafting residues 19–24 from 4ERF [30] (resolved at 2.0 Å) on to 1YCR. This yielded a final HDM2 with residues 19–109 of human HDM2; the p53 segment from residues 17–29 as found in 1YCR was used and Nutlin from 1RV1 was used. The N- and C- termini of were capped with acetyl (ACE) and N-methyl (NME) respectively to keep them neutral. Molecular dynamics simulations were performed with the SANDER module of the AMBER11 [31] package employing the all-atom Cornell force field [32]. Nutlin parameters were built using antechamber [33]. All systems were prepared as described before [23] and simulated for 100 ns at constant temperature (300 K) and pressure (1 atm) and structures were stored every 1ps. The free energies of binding (ΔG_bind_) of the p53 and Nutlin to HDM2 were computed and visualizations were carried out as described earlier [23].

Interactions between the acidic domain of HDM2 and the DNA binding domain (DBD) of p53:

The acidic domain of HDM2 is known to be unstructured [34], and hence we used molecular dynamics in implicit solvent (AMBER molecular modeling package) [31] to model a 24-residue peptide in the acidic domain (residues 259–282 of HDM2) starting from an extended conformation. These structures were then used as input into the program HADDOCK [35], which docks molecules based on geomteric restraints between two sets of “active” residues. The “active” residues are chosen based on available experimental data. For HDM2, a region in the acidic domain (residues 260, 262, 270, 271, 272, 273, 274, 276, 277) was selected as active while for the p53 protein, a monomer of the core domain in the absence of DNA (PDB id: 2OCJa) was used, and the active residues considered to be important for peptide binding [35] were chosen (residues 114, 115, 117, 118, 279, 280, 282, 283, 286, 248). These restraints were then processed and structures optimized by HADDOCK. The obtained complexes were further refined using Rosetta's FlexPepDocking protocol [36], ensuring that they remained consistent with the experimental restraints. The same procedure was repeated for the V280A mutant.

## Results

### *In vitro* Selection of Nutlin-resistant HDM2

**Figure 1:**
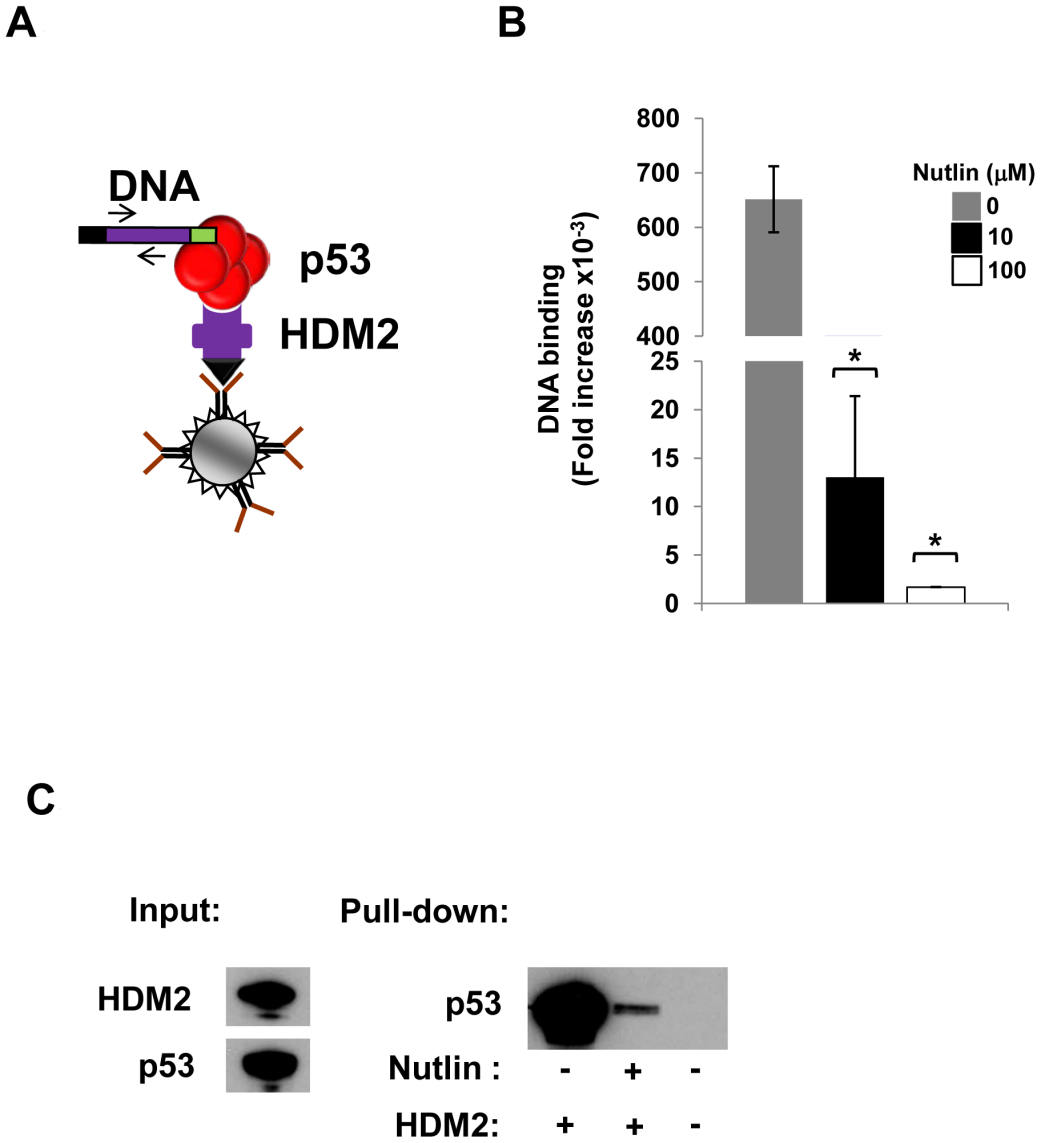
*In vitro* detection of HDM2-p53-DNA complex and disruption by Nutlin. A, Schematic depicting complex formed between HA-tagged HDM2, p53 and DNA captured on beads coated with anti-HA antibody. The HDM2 expression construct (purple bar) comprises HA-tag encoding sequence (black) and p53 RE (green). Arrows depict PCR primers to quantify captured DNA. B, Real-time PCR assay to measure complex formation (shown in A) in the presence of Nutlin (0,10,100 µM). Values indicate fold increase over HDM2 gene control without any p53 response element (2CONA) appended. Values represent mean ± SD (n = 2), *p<0.05. C, Western blot of p53 captured by immobilised HDM2 and effect of Nutlin (10 µM).

A previous IVC selection for p53 variants with altered binding specificities linked genotype with phenotype by p53 binding back to the gene encoding it via an appended p53 DNA response element (RE) [24]. To enable selection of HDM2 variants capable of binding p53 in the presence of Nutlin, we first determined whether an HDM2-p53-DNA complex (Figure 1A) is able to form *in vitro*. HA-tagged HDM2 was expressed *in vitro* from a DNA template to which two copies of the p53 CONA response element [37] were appended. Magnetic beads coated with anti-HA antibody were added to the *in vitro* reaction to capture the HDM2 protein, following which p53 protein (also expressed *in vitro*) was added. After incubation, the beads were washed and DNA captured on the beads quantified by real-time PCR. A control reaction was also carried out wherein the DNA template encoding HDM2 did not have the 2CONA RE appended. The results show that DNA is only captured on the beads when the 2CONA RE is present, indicating the formation of an HDM2-p53-DNA complex (Figure 1B). Importantly, addition of Nutlin resulted in a clear dose-dependent reduction in the amount of 2CONA-appended DNA pulled down (∼ 383 fold reduction at 100 µM), indicating disruption of the p53-HDM2 interaction *in vitro*. Disruption was also observed in a pull-down assay measuring p53 bound to immobilised HDM2 by Western blot (Figure 1C).

**Figure 2:**
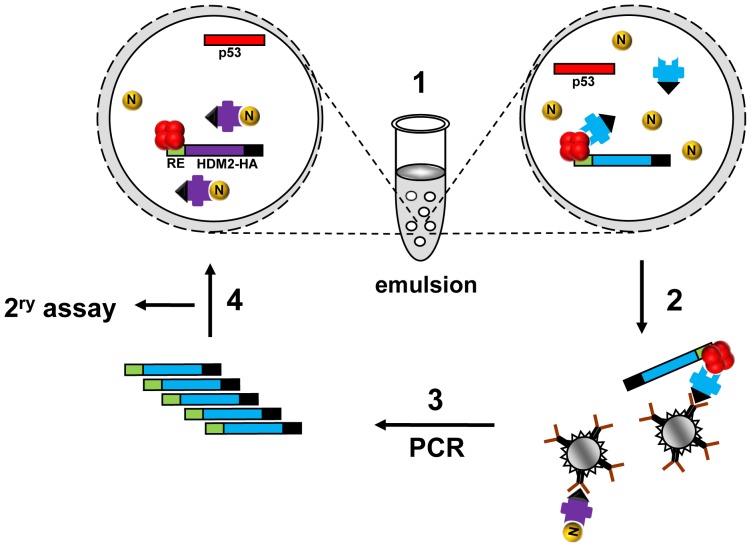
Selection of Nutlin-resistant HDM2 by *in vitro* compartmentalisation. 1, HDM2 expression constructs (blue and purple bars) appended with 2CONA p53 response element (green) and HA-tag coding sequence (black) and p53 expression construct (red bar) are segregated into aqueous emulsion compartments along with Nutlin (yellow orb). Protein expression occurs within compartments. Nutlin inhibition of HDM2 results in no HDM2-p53-DNA complex formation (left bubble), whereas resistant HDM2 can form the complex (right bubble). 2–3, The emulsion is broken and complexes captured with anti-HA antibody. DNA encoding resistant HDM2 variants is amplified by PCR. 4, Selectants further evaluated by secondary pull-down assay or subjected to further rounds of selection.

**Figure 3:**
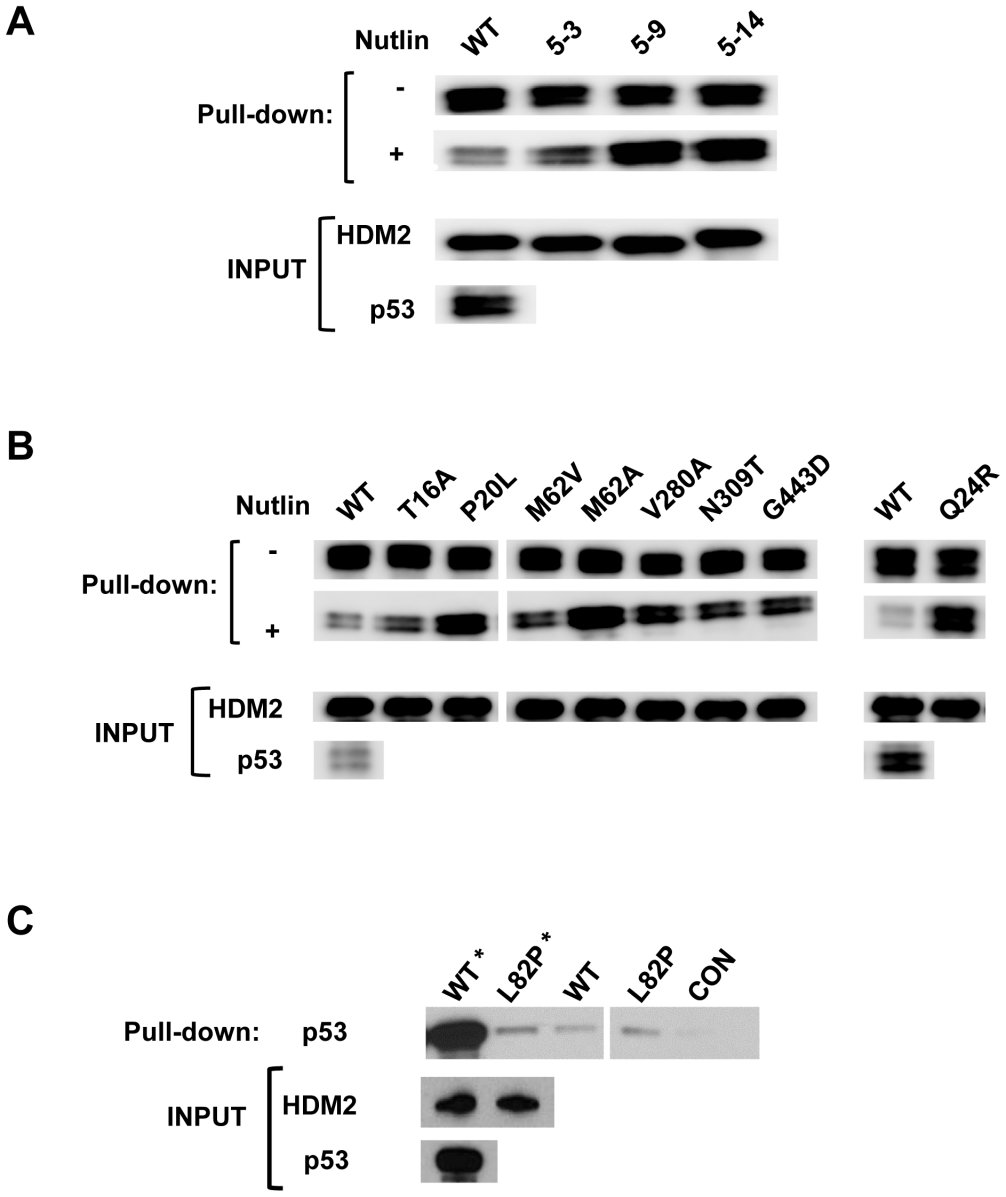
Selected HDM2 variants display *in vitro* Nutlin-resistance phenotype. A,B *In vitro* pull-down assay showing reduced inhibition by Nutlin (10 µM) to binding of p53 for indicated parental HDM2 variants and point mutants derived from parental clones (5.9 and 5.14 respectively). 50% of p53 protein pulled down in absence of Nutlin treatment loaded. C, Analysis of L82P mutation indicates reduced inhibition by Nutlin (100 µM). Control indicates background p53 binding in absence of HDM2. * Indicates no Nutlin treatment.

**Figure 4:**
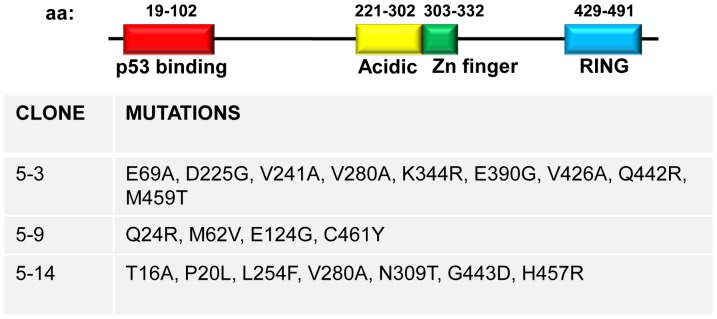
Domain architecture of HDM2 gene. Mutations present in HDM2 selectants displaying *in vitro* Nutlin resistance are shown in table below.

Based on these results, we created a library of randomly mutated HDM2 genes and carried out selection for Nutlin-resistance by IVC. Figure 2 depicts the selection protocol, wherein variant HDM2 expression constructs tagged with the CONA RE, along with p53 expression construct and Nutlin are dispersed into the aqueous compartments of the water-in-oil emulsion. Within each compartment protein expression occurs, and in the presence of Nutlin, the HDM2-p53-DNA complex is not expected to form if Nutlin binds HDM2 (left bubble), but will form if the variant HDM2 is resistant to Nutlin inhibition (right bubble). After formation of complexes, the emulsion is broken and complexes are captured using anti-HA coated magnetic beads. The genes encoding Nutlin-resistant HDM2 variants are then amplified by PCR prior to further rounds of selection and/or secondary characterisation. After 5 rounds of selection, 15 clones were analysed in a secondary pull-down assay. Of these, 3 showed significantly more binding to p53 in the presence of Nutlin compared to wild-type HDM2 (Clones 5.3, 5.9, 5.14; Figure 3A, Figure S1 in File S1). Sequence analysis indicated several mutations in the N-terminal p53/Nutlin binding domain (amino acids 19–102) [29], [38]. Additionally, mutations were seen in the central acidic (amino acids 221–302) [39] zinc-finger (amino acids 297–329) [40] and RING (amino acids 429–491) [41] domains (Figure 4). Investigation of the individual contribution of each mutation indicated that T16A, P20L, Q24R, M62V (N-terminal domain), V280A (acidic domain), N309T (zinc finger domain) and G443D (RING domain) in isolation conferred Nutlin resistance (Figure 3B, Figure S1 in File S1). The recently described Nutlin-resistant M62A mutant [42] was included as a positive control. Apart for the V280A mutation in HDM2-5.3 (also present in HDM2-5.14), the remaining mutations in this clone did not display significant resistance when assayed in isolation (Figure S2 in File S1). It is possible that these mutations are epistatic for the resistance phenotype. In the absence of Nutlin, binding of the HDM2 point mutants to p53 was not significantly different from wild-type (Figure 3B, Figure S1 in File S1).

Sequence analysis of round 5 selectants showed the mutation L82P in the N-terminal domain to occur in two independent clones. Analysis of HDM2 harbouring this point mutation indicated a marked decrease in binding to p53 (Figure 3C). However, Nutlin had no effect on this binding, indicating significant resistance which was recapitulated in a cell-based assay (see below).

### *In vitro* Selectants Display Nutlin-resistant Phenotype in Functional Cell Assay

**Figure 5:**
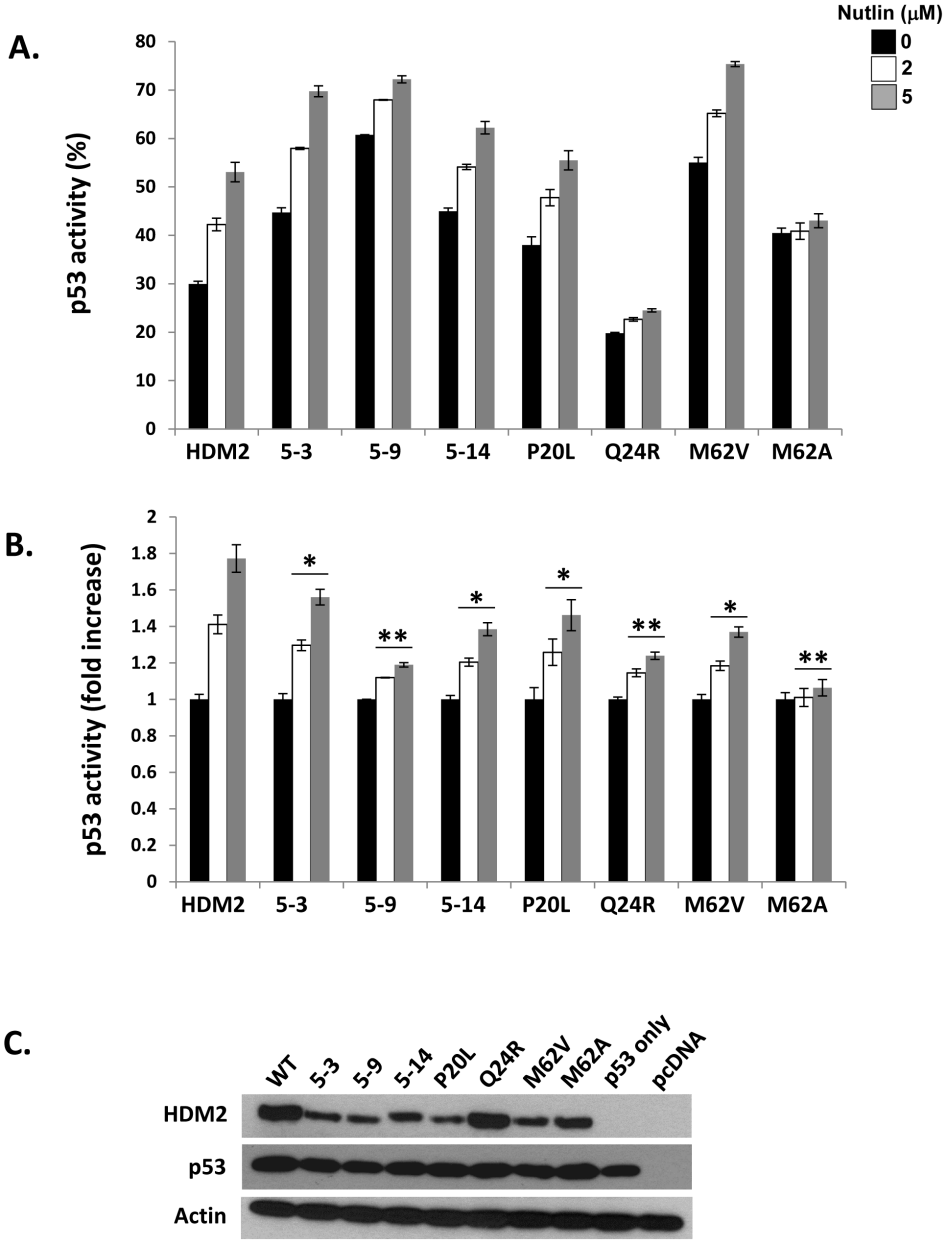
Nutlin shows reduced inhibition of selected variants in p53-null H1299 cells. A, H1299 cells transfected with either p53 alone or p53 and indicated HDM2 variants. P53 function measured by reporter gene activity in presence of Nutlin (0,2,5 µM). Values represent mean ± SD, n = 2. B, Same as in A, with p53 activity shown as fold-increase over the base-line value of inhibition in the absence of drug treatment (set to 1). Values represent mean ± SD, n = 2. *p<0.5, **p<0.05. C, Western blot indicating expression levels of HDM2 variants and p53 in H1299 cells.

The parental selectants and the single HDM2 mutants Q24R, P20L and M62V were next analysed in a functional assay measuring p53 activity in the H1299 cell line (Figure 5A). The M62A mutant was also included as it has not been previously characterized in cell-based reporter assays. Plasmids encoding HDM2 (wild-type or selectants) and p53 were transfected along with a p53 transactivation reporter construct. In the presence of Nutlin, inhibition by HDM2 was attenuated, with p53 activity being restored up to 53% of that observed in the absence of HDM2 co-transfection (5 µM Nutlin). HDM2 Q24R showed an appreciable Nutlin-resistant phenotype, with p53 activity only being restored to 24% (5 µM Nutlin). The M62A mutant also showed reduced p53 activity (43% at 5 µM Nutlin).

Whilst the parental clones and the mutants P20L and M62V did not show a net resistance phenotype, Nutlin was clearly less effective on these mutants when p53 activity was compared to the basal value of inhibition in the absence of drug (Figure 5B). This value was elevated for all the parental clones, most likely due to their reduced expression levels compared to wild-type HDM2 (Figure 5C), particularly selectant 5.9.

**Figure 6:**
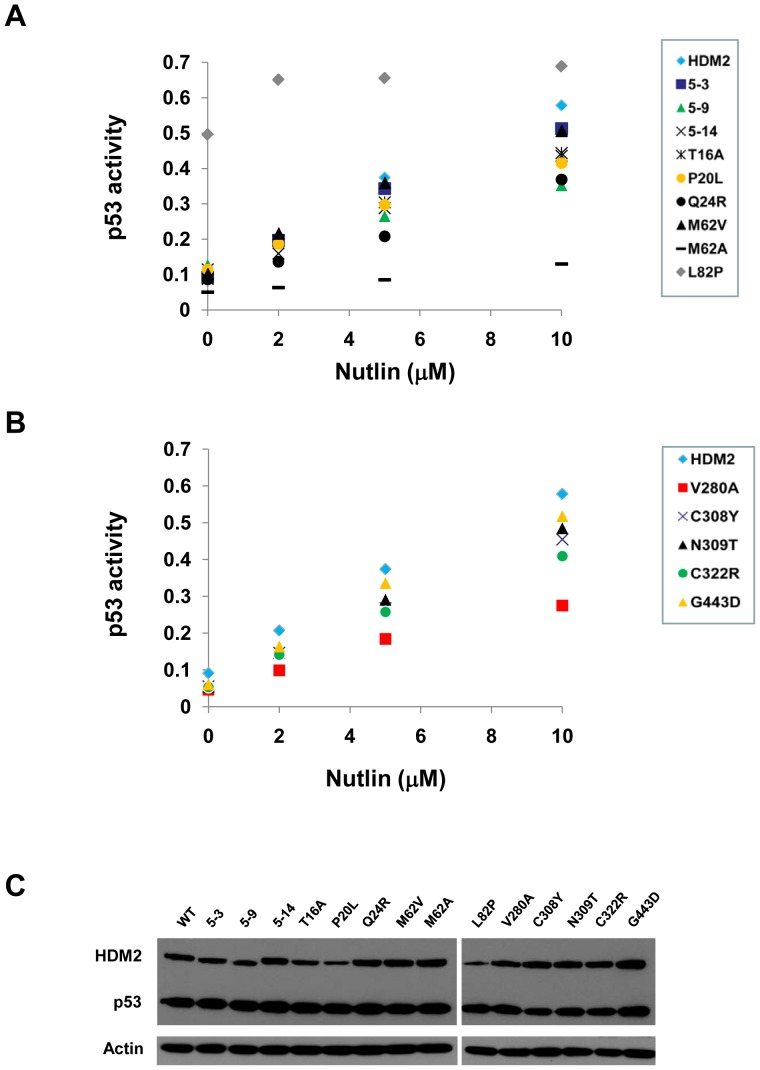
Nutlin shows reduced inhibition of selected HDM2 variants in p53/MDM2-null DKO cells. A, Wild-type and indicated N-terminal domain mutants were co-transfected with p53 and p53 reporter gene activity measured in the presence of Nutlin (0,2,5,10 µM). At the highest dose, wild-type HDM2 displays ∼29% reporter activity compared to p53-alone transfection. Representative data from one experiment shown; see Figure S3 in File S1 for replicate experimental data and statistical analyis. B, Wild-type and indicated acidic (V280A), zinc finger (C308Y, N309T, C322R) and RING (G443D) domain mutants were co-transfected with p53 and p53 reporter gene activity measured in the presence of Nutlin (0,2,5,10 µM). Representative data from one experiment shown; see Figure S3 in File S1 for replicate experimental data and statistical analyis. C, Western blots indicating expression levels of HDM2 variants and p53 cotransfected into DKO cells.

As these results were possibly impacted on by the presence of endogenous HDM2 in H1299 cells, we repeated the assay in the p53/MDM2-null DKO cell line [43] (Figure 6, Figure S3 in File S1). In this cell line, the parental HDM2-5.9 and 5.14 selectants displayed a resistance phenotype at all Nutlin doses tested, as shown by the reduced p53 activation compared to wild-type HDM2. Analysis of individual mutations indicated Q24R, P20L, and T16A in the N-terminal domain to elicit moderate resistance phenotypes, all showing between 50–80% restoration of activity seen with wild-type HDM2. M62V did not display any significant resistance. Despite the comparatively lower expression levels of the L82P mutant it was still able to abrogate p53 activity by ∼66% in the absence of Nutlin (Figures 6A,C). As with the *in vitro* pull-down assay, the L82P point mutant was highly Nutlin resistant, with very little restoration of p53 activity at the highest dose of Nutlin (∼1.5-fold increase compared to ∼6,4-fold increase seen for wild-type HDM2). We also included the M62A mutant, previously shown only by *in vitro* pull-down to be Nutlin resistant [42]. This mutant showed strong resistance in this assay, with p53 activity only being restored to ∼30% of that seen with HDM2. Within the acidic domain, V280A showed ∼55% activity of wild-type (10 µM Nutlin). The N309T mutant in the zinc finger domain showed slight resistance (∼88% activity of wild-type, 10 µM Nutlin). However, its proximity to C308, shown to be mutated in non-Nutlin treated cancer [19] led us to test the clinically observed C308Y mutant and this showed moderate resistance (∼70% activity of wild-type, 10 µM Nutlin). The C322R mutation in the zinc finger domain also showed moderate resistance (∼73% activity of wild-type, 10 µM Nutlin ). The G443D mutant in the RING domain showed slight resistance (∼85% restoration, 10 µM Nutlin).

**Figure 7:**
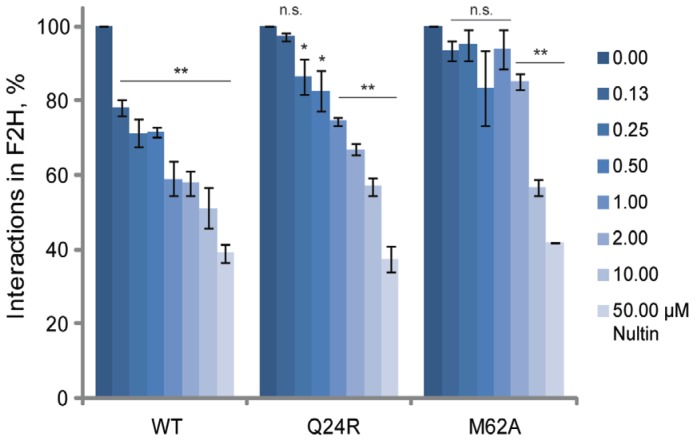
Nutlin shows reduced inhibition of the direct interaction between p53 and HDM2 N-terminal domain. F2H assay to investigate the interaction of p53 (bait) with wild-type (WT) HDM2, mutant Q24R and M62A (preys). The F2H assay measures the interaction between two proteins as ratio of cells showing co-localization of bait and prey at the nuclear F2H interaction platform, to cells not showing this co-localization. Titration of Nutlin on to BHK cells co-transfected with GFP-p53 and RFP-HDM2 (wt) immediately shows declined percentage of co-localization. In contrast, p53 interaction with HDM2 mutants Q24R and M62A upon Nutlin treatment is clearly less reduced, indicating Nutlin resistance. Graph bars show means of normalized interaction values (in %) ± s.e.m. from three to five independent experiments. n.s. no significance, *p<0.05, **p<0.01.

We further characterized the direct cellular binding of the HDM2 wild-type, Q24R and M62A N-terminal domains to p53 in the Fluorescent 2-Hybrid (F2H) assay [28]. The F2H assay differs from the DKO reporter assay, as it does not measure reactivation of a reporter gene but the precise interaction to be disrupted. The assay visualizes the interaction of RFP-tagged HDM2 (amino acids 7–134) with GFP-tagged p53 (amino acids 1–81) at a defined nuclear F2H interaction platform, in specific BHK cells. The addition of Nutlin results in a dissociation of the complex, which can be imaged and quantified. Compared to the wild-type HDM2-p53 interaction, addition of Nutlin resulted in reduced dissociation of mutant N-terminal domains from p53, indicating Nutlin resistance (Figure 7). For wild-type HDM2, a highly significant reduction of interactions was measured at 0.13 µM Nutlin. For the two mutants Q24R and M62A, no significant reduction was detectable until addition of at least ten times higher concentrations of Nutlin (1 and 2 µM respectively). The Q24A interaction with p53 was disrupted less significantly in the range of 0.25–1 µM Nutlin. M62A clearly showed a stronger phenotype than Q24R, which is in accordance with the reporter assay in DKO cells. Furthermore, time-lapse analysis indicated enhanced persistence of mutant HDM2-p53 complexes compared to wild-type after Nutlin challenge (1 µM). The wild-type complex was not visible after 20 minutes, whilst the Q24R complex lasted for 40 minutes. The M62A complex was still visible after one hour (Figure S4 in File S1, video in Figure S6).

## Discussion

We have used a completely *in vitro* selection platform to evolve Nutlin-resistant variants from a large repertoire (∼10^9^) of randomly mutated HDM2 genes. These variants harboured multiple mutations throughout all the domains comprising HDM2. As acquired drug resistance can arise through point mutations [44], [45], we analysed the mutations in isolation, and several of these displayed the Nutlin-resistant phenotype both *in vitro* and *ex vivo*.

Residues 16–24 in the p53 binding domain of apo HDM2 comprise a flexible lid region shown to behave as a weak pseudo-substrate in the absence of p53 binding [46], [47]. NMR studies indicate that whilst the lid predominantly adopts the “open” conformation when p53 is bound, Nutlin-binding is compatible with both the “open” and “closed” lid-binding states [48]. Hence the mutations T16A, P20L and Q24R may further weaken this intra-molecular interaction to selectively increase the interaction with p53. Whilst the pull-down experiments (Figure 3B) did not show any major differences in binding, a more accurate determination of relative affinities is required to fully investigate this hypothesis. In support of this model, biochemical studies show the phosphomimetic mutation S17D in the lid to stabilize the HDM2-p53 interaction [49], [50]. A similar model, suggested by molecular simulations of the complexes of HDM2 (with lid) and p53/Nutlin indicates that P20 makes weak interactions with the hydrophobic side chains of L26 and P27 of p53 (manuscript in preparation). Mutation to the more hydrophobic leucine is predicted to selectively enhance these interactions which are absent when Nutlin is the ligand.

**Figure 8:**
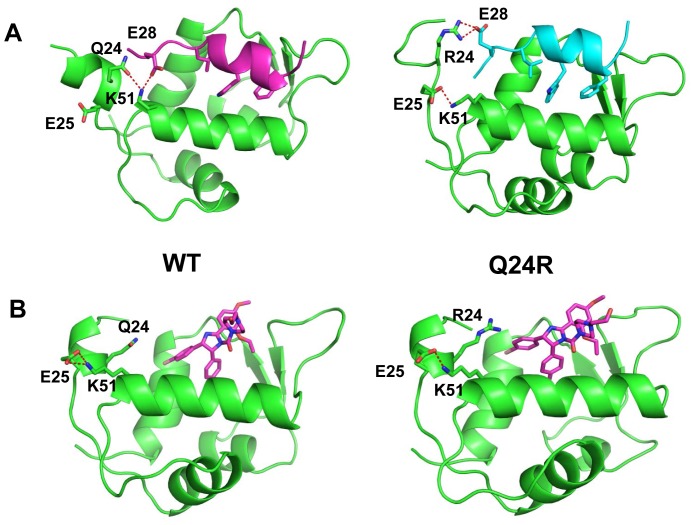
The Q24R mutation in the HDM2 lid region is predicted to enhance affinity for p53 but not Nutlin. A, Molecular simulations (detailed in Materials and Methods) indicate mutation of Q24 to arginine (right structure) leads to repulsion of proximal K51 and stabilization of E28 in p53 (amino acids 17–29, blue) through charge-charge interaction. This additional stabilization is not seen in wild-type HDM2 bound to p53 (left structure, p53 shown in purple). B, Q24 makes no significant contacts with Nutlin (left structure) and mutation to arginine (right structure) makes no additional difference.

Studies have shown that K51 of HDM2 interacts with E28 of p53 [34]. Simulations indicate that the Q24R mutation leads to the development of a cationic potential in the region of R24. This results in repulsion of K51, which in turn stabilizes anionic E28 of p53 through a charge-charge interaction and enhances the affinity of p53 for HDM2 (Figure 8A). In the presence of Nutlin, no such interaction is possible, thus R24 remains solvent exposed (Figure 8B). The energetics of binding further reflect this trend (Figure S5 in File S1), and this mutant appears to confer resistance by stabilizing p53 binding without affecting Nutlin binding. A similar mechanism has been described for a point mutant of the EFGR kinase which causes resistance to the ATP-analogue gefitinib by increasing affinity for ATP [45].

The L82P mutation in the N-terminal domain conferred significant Nutlin-resistance, although this came at a cost of greatly reduced binding to p53 *in vitro* and reduced stability/expression *ex vivo*. L82 lies within the α1′ helix forming part of the floor of the hydrophobic p53-binding pocket. The lower expression level of this mutant in cells indicates that substitution to the less hydrophobic proline places constraints on the secondary structure and destabilizes the overall N-terminal domain fold. Conformational changes associated with this destabilization could preferentially abrogate Nutlin binding as it makes fewer contacts with HMD2 than p53 [9], [29]. Additionally, p53 binding could preferentially stabilize this mutant by shielding otherwise solvent-exposed hydrophobic residues. Whilst this mutation confers significant resistance to Nutlin, the overall loss of activity due to reduced stability makes it less likely to occur in cancer. Further stabilizing mutations and/or gene duplication with attendant increases in expression levels could however permit the emergence of this and related mutations in cancer.

**Figure 9:**
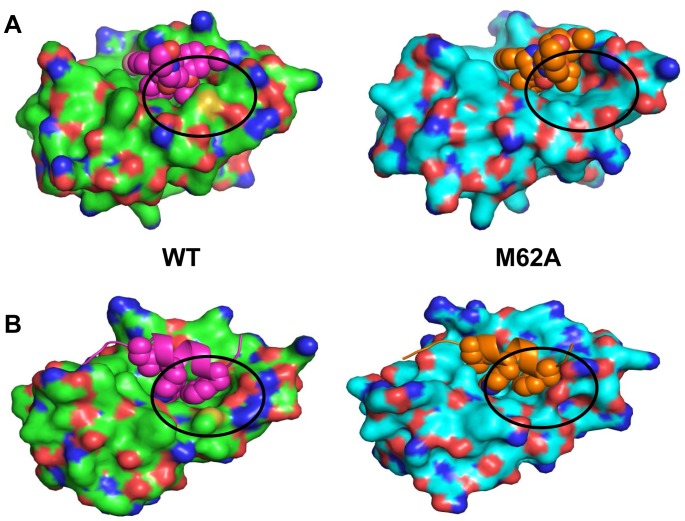
The M62A mutant in the HDM2 p53-binding domain selectively results in loss of Nutlin binding. A, Simulations (see Materials and Methods) indicate loss of significant packing interactions with Nutlin (left structure, circled) when M62 is mutated to alanine (right structure, circled). Nutlin respectively depicted in purple and orange. B, Packing interactions with p53 (left structure, circled) are minimally disrupted by M62A mutation (right structure, circled). p53 (amino acids 17–29) respectively depicted in purple and orange.

Selection of the M62V mutation was of particular interest, as we have previously shown M62A to confer Nutlin resistance *in vitro* [42]. The amino acid M62 is an essential part of the subpocket accommodating F19 of p53 in the HDM2-p53 interaction [29], and small molecules such as Nutlin designed to mimic the three key interactions (F19, W23 and L26) of p53, also interact with this subpocket [23]. M62 makes direct contacts with both p53 and Nutlin in their respective crystal structures [9], [29]. However, the mutation M62A causes the loss of a significant fraction of packing interactions with Nutlin, thus selectively destabilizing its binding with less impact on p53 which makes several additional contacts with HDM2 over an extended surface (Figure 9) Again, the energetics of the interactions show that binding of Nutlin is destabilized (Figure S5 in File S1) while that of p53 is marginally affected, thus providing a mechanism for the observed resistance. Mutation of methionine to alanine would require mutagenesis of two adjacent nucleotides (AT to GC), which is unlikely to occur using the error-prone PCR mutagenesis employed in this study. However mutation to valine, one of the more similar amino acids to alanine, required only a single base change. The weaker *ex vivo* phenotype of M62V compared to M62A suggests that mutation to valine impacts negatively on post p53-binding events described below.

**Figure 10:**
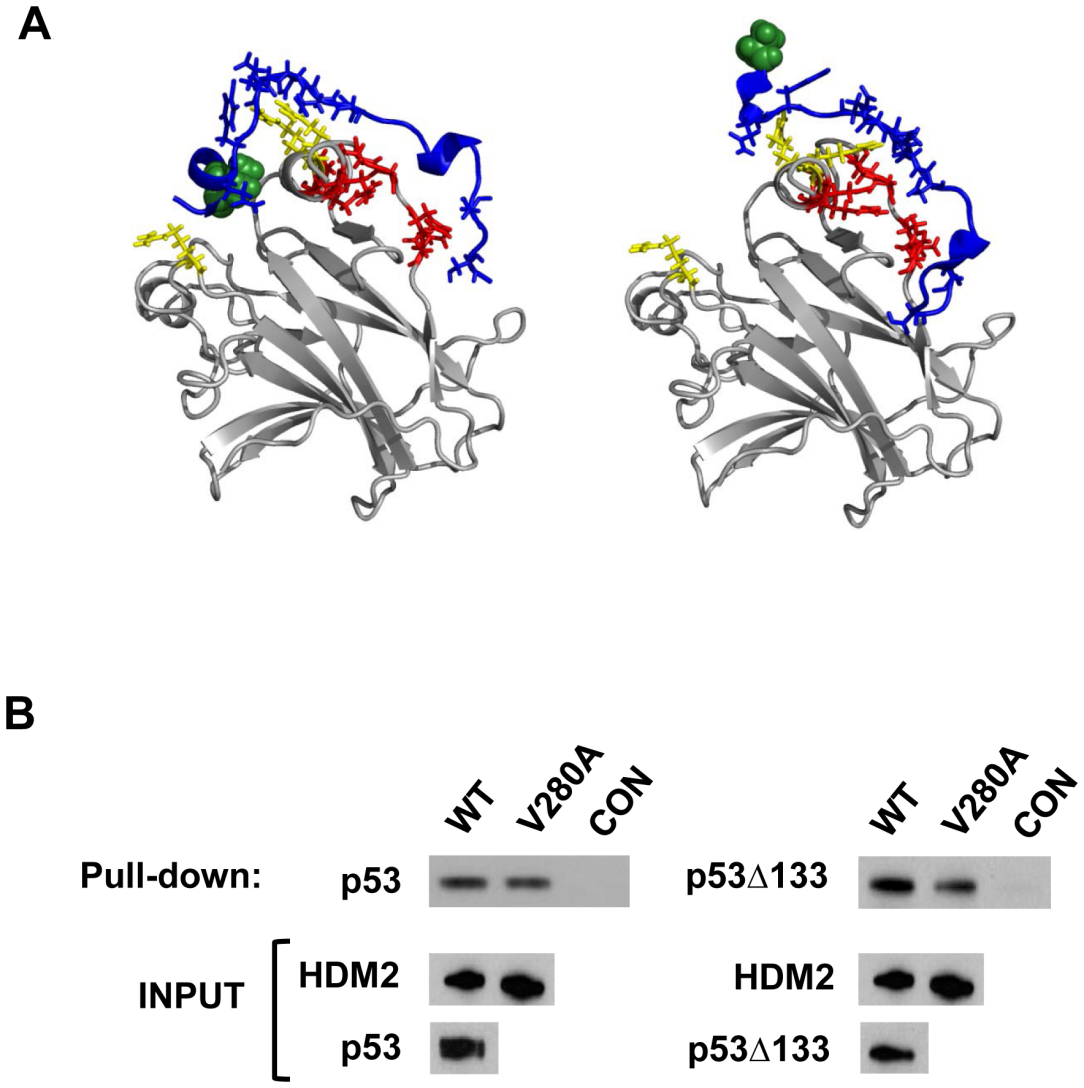
Mutation V280A in acidic domain results in reduced interaction with p53 DNA binding domain. A, Models of the native (left) and V280A mutant (right) peptides docked to the p53 DNA binding domain. Residues set as “active” in the Haddock server run (details in Materials and Methods) are shown as sticks (red and yellow for p53 and blue for peptide). Residues of p53 known to make direct contact with DNA are colored in yellow. V280 and A280 are shown in green in the respective structures. In the native case V280 appears to make more contacts with p53 than A280 in the mutated peptide. B, *In vitro* pulldown assay shows reduced interaction between HDM2-V280A and N-terminally truncated p53 (Δ133, comprising secondary HDM2 interaction site only). Interaction with full-length p53 (comprising both N-terminal and secondary HDM2 interaction sites) is unaffected.

The central acidic domain of HDM2 contains a secondary binding site that interacts with the p53 DNA binding domain [34], [51]. Mechanistically, it could be expected that mutations in this region that increase the secondary interaction might be selected to counteract Nutlin-induced loss of the primary interaction site. However, for the V280A mutation, which lies within the secondary binding site, *in silico* prediction suggested the converse, and this was subsequently verified by *in vitro* pulldown (Figure 10). This points to an allosteric mechanism, as previously shown for mutations in the HDM2 C-terminal RING domain, that impact on Nutlin binding [52]. Such a mechanism could further account for the other mutations identified in this study that lie outside the HDM2 p53-binding domain. The central regions of HDM2 additionally interact with negative regulators including ARF and RPL11 [53], [54]. Notably, cancer-associated mutations including C308Y in the central zinc finger domain have been described (in non-Nutlin treated individuals) which disrupt interaction with RP11 [55]. Guided by the *in vitro* selection of the N309T zinc finger domain mutation, we investigated the C308Y mutation, and this conferred Nutlin-resistance. Hence, there is precedence for future clinical resistance arising through mutations in the central acidic and zinc finger domains, which concurrently inhibit binding of both Nutlin and regulatory proteins.

Our data indicates that resistance to Nutlin can arise through several mechanisms. In the case of the N-terminal domain HDM2 mutants, these are predicted to either selectively reduce affinity for Nutlin (M62A, M62V, L82P) increase affinity for p53 (P20L, Q24R) or influence lid dynamics (T16A, P20L, Q24R). These effects can possibly be overcome by designing small molecule derivatives capable of forming additional contacts with the HDM2 binding pocket. Recently described examples include a series of piperidinones, which in addition to the three core p53-mimetic interactions, form additional Van der Waals, pi-stacking and electrostatic interactions with HDM2 [30]. Alternatively, stapled-peptide derivatives of the p53 motif that interact with HDM2 should prove more recalcitrant to mutation by virtue of the increased interaction footprint [10], [56], [57] and experiments indicate this to be the case (manuscript in preparation). The absence of structural data for full-length HDM2 makes it difficult to understand probable allosteric effects of mutations outside the N-terminal domain that impact on Nutlin binding (V280A, C308Y, N309T, C322R, G443D). However, C-terminal RING domain mutants have been described which increase the affinity of the HDM2-p53 interaction [52]. Therefore, as with N-terminal domain mutations that increase p53-binding, the use of small molecules and stapled peptides with increased binding footprints may offset allosterically induced structural variation and compete more efficiently with p53 for binding.

It is desirable to increase selection pressure during rounds of directed evolution, and in the present study we were restricted to some extent by the low solubility limit of Nutlin and its strong hydrophobicity [58], which most likely led to much of it partitioning into the oil phase of the emulsion. Despite this, enough selection pressure was applied to yield several clones harbouring multiple mutations which showed the desired phenotype. Some discrepancy was however observed between the two assay formats. For example, HDM2-5.3 showed appreciable binding to p53 in the presence of Nutlin in the *in vitro* pull-down assay, but displayed a mild phenotype in the *ex vivo* functional assay. The first assay measures binding of HDM2 to p53, whilst the second is the aggregate readout for inhibition of p53 activity arising from the binding, inhibition of transactivation, and E3 ligase activites of HDM2. Hence mutations ancillary to V280A in selectant 5.3 (which confers Nutlin resistance in isolation) likely impact negatively on the latter two activities in the *ex vivo* assay. The V280A mutation is also present in selectant 5.14 which shows essentially the same phenotype in both assays, indicating context-dependency. Overall, the assumption that *in vitro* binding can be used as a proxy to measure HDM2 function in the cell-based assay is validated through selection of HDM2 variants 5.9 and 5.14 which behave similarly in both assays.

The experimental approach to anticipating cancer drug resistance has most commonly involved the treatment of drug-sensitive cell lines, followed by analysis of resistant subpopulations [59]. Non-targeted *in vitro* mutagenesis of a cell line, followed by treatment and selection for resistance has also been described [60]. Target-based mutagenesis approaches, wherein complementation by a mutated protein enables survival of an otherwise drug-sensitive cell line, have correctly anticipated drug resistance [61], [62]. However, a major disadvantage is the relatively small library of variants that can be sampled due to inherent technical limitations (∼10^6^), and the possibility of off-target drug toxicity at higher doses limiting selection pressure. By comparison, IVC readily enables interrogation of up to 10^10^ variants [63] and generally allows for application of stringent selection pressures (although in this particular case the physicochemical properties of Nutlin were limiting). Being completely *in vitro*, IVC may not be suitable where the function of target proteins requires post-translational modification, and for certain targets it may not be trivial to devise a selection strategy. However, where *in vitro* selection is possible, a robust approach to modeling drug resistance could entail primary use of IVC to sample a large pool of diversity for mutation hotspots. Smaller, focused libraries covering these regions could then be generated, and these further analysed in cell-based complementation assays.

## Supporting Information

File S1 **Supporting information Figure S1 to S6.**
**Figure S1 Selected HDM2 variants display in vitro Nutlin-resistanc phenotype.** A, in vitro pull-down assay showing reduced inhibition by Nutlin (10 µM) to binding of p53 for indicated parental HDM2 variants. Data represents binding of p53 (determined by densitometric analysis after Western blotting of 2 independent experiments) to each variant expressed as a percentage of that observed in the absence of Nutlin treatment. Values represent mean ± SD. B, as in A except for indicated point mutants. C, in vitro pull-down assay showing binding of HDM2 and variants to p53 in absence of nutlin. Binding of mutants (determined by densitometric analysis after Western blotting of 2 independent experiments) is expressed relative to binding of wild-type HDM2 to p53 (set to 100%). Values represent mean ± SD.
**Figure S2 Effect of Nutlin on p53-binding for HDM2 variants carrying single mutations derived from parental clone HDM2-5.3.** In the presence of Nutlin (10 µM), no significant increase in p53 binding is observed for mutant HDM2 compared to wild-type.
**Figure S3** **Nutlin shows reduced inhibition of selected HDM2 variants in p53/HDM2-null DKO cells.** DKO cells co-transfected with p53 and indicated HDM2 variants. p53 function measured by reporter gene activity in presence of indicated amounts of Nutlin. Activity expressed as percentage of reporter gene transactivation seen with wild-type HDM2 (set to 100, indicated by dotted line). Values represent mean ± SD from two to three independent experiments, *p<0.5, **p<0.05, ***p<0.005.
**Figure S4 Time lapse images indicate persistence of mutant HDM2-p53 complex in presence of Nutlin using F2H assay.** Green dot shows p53 bound to DNA. Co-localised red dot shows HDM2 in complex with p53. Arrows indicate last time point where HDM2 (wild-type or indicated mutant) is present in complex. Time is indicated in minutes.
**Figure S5** **The distribution of energies of interactions (enthalpies) of p53 (top) and Nutlin (bottom) with wild-type and the mutants Q24R and M62A.** The enthalpies are computed using standard protocols as outlined earlier [23].
(PDF)

Video S1 **Video footage of F2H assay measuring interaction between HDM2 (wild-type and indicated mutants) and p53 indicates persistence of mutant HDM2 interactions with p53 compared to wild-type.** Green dot shows p53 bound to DNA. Co-localised red dot shows HDM2 in complex with p53. Time is indicated in minutes.
(PPTX)
