# Supplementary figures and images for: In Vitro Selection of Mutant HDM2 Resistant to Nutlin Inhibition
Source: PLoS One. 2013 Apr 30;8(4):e62564. doi: 10.1371/journal.pone.0062564 (PMC3641235; doi:10.1371/journal.pone.0062564)

Figure S1

A.

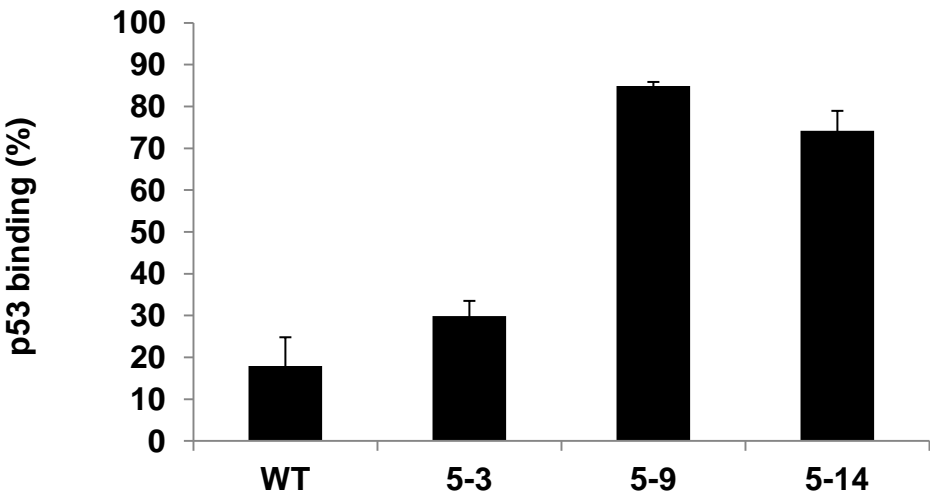

B.

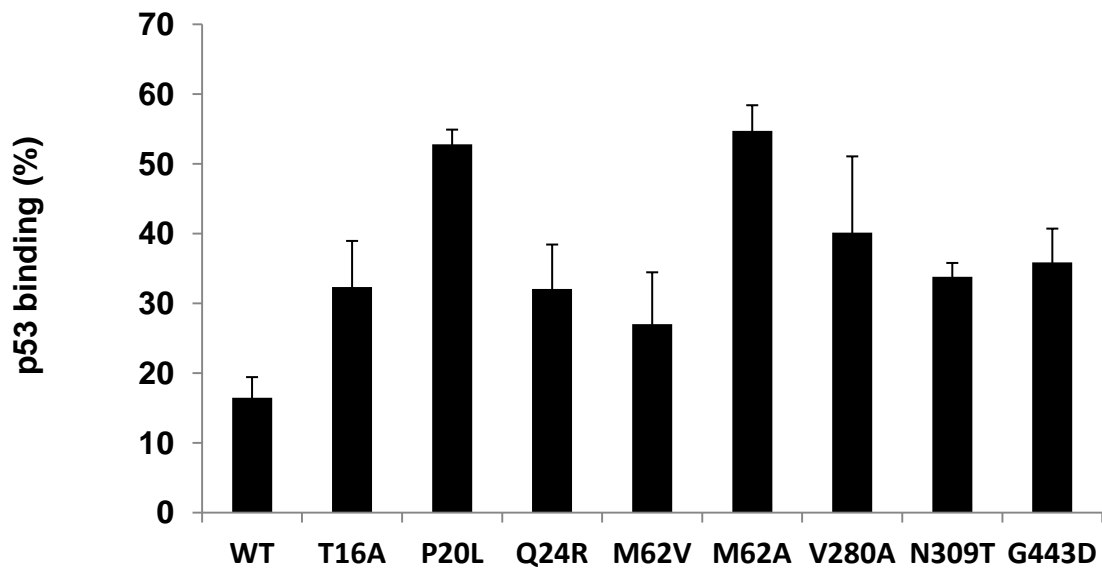

C.

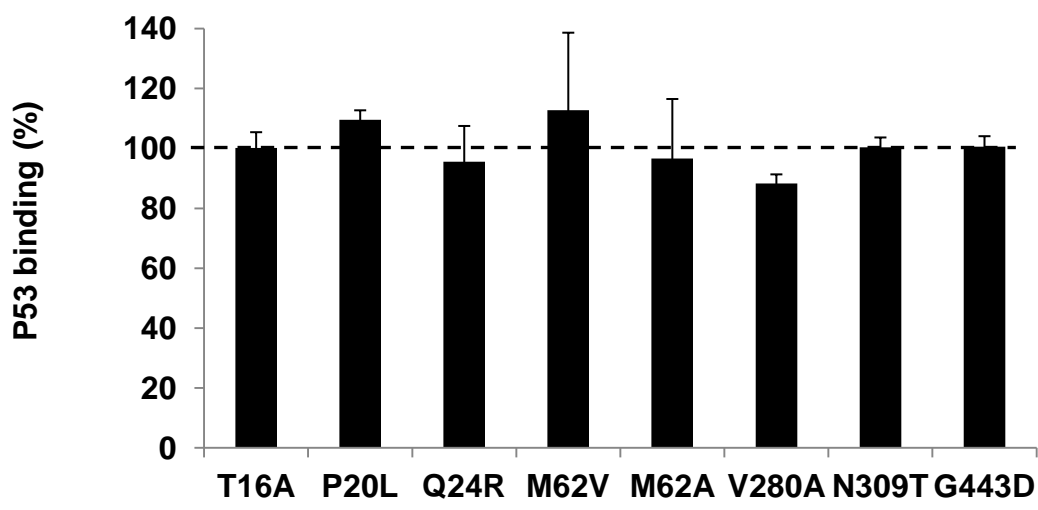

Figure S2

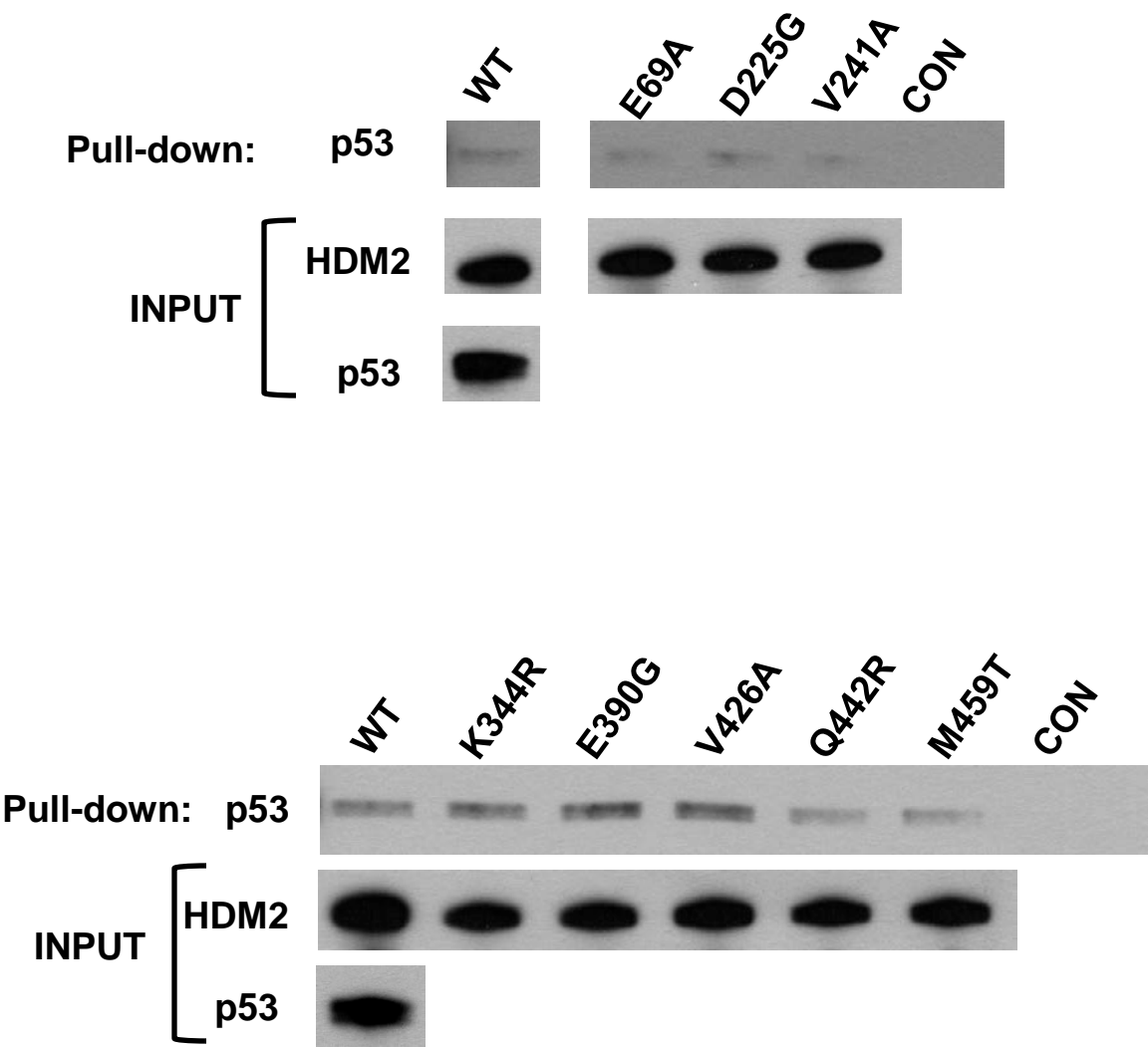

Figure S3

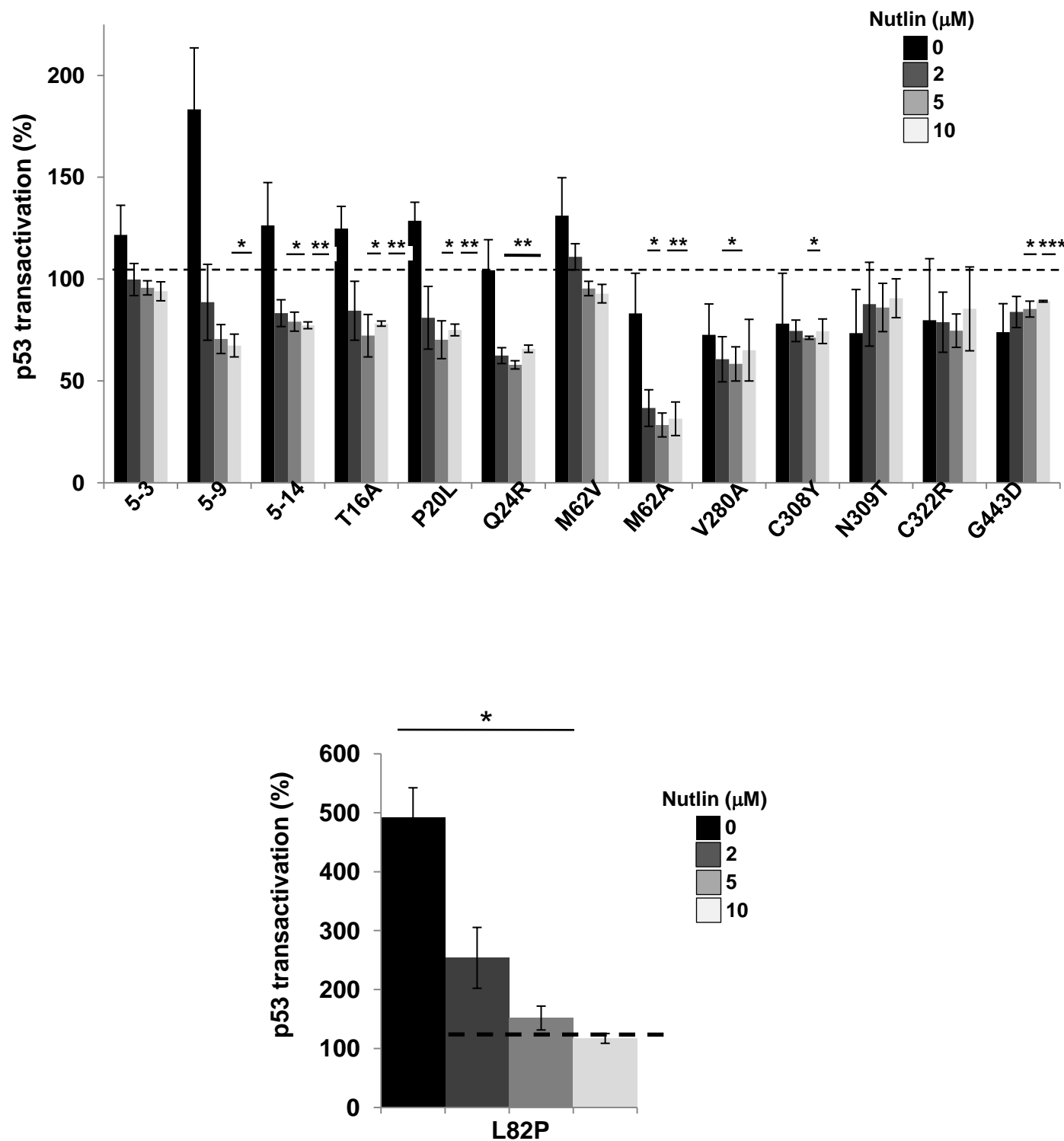

Figure S4

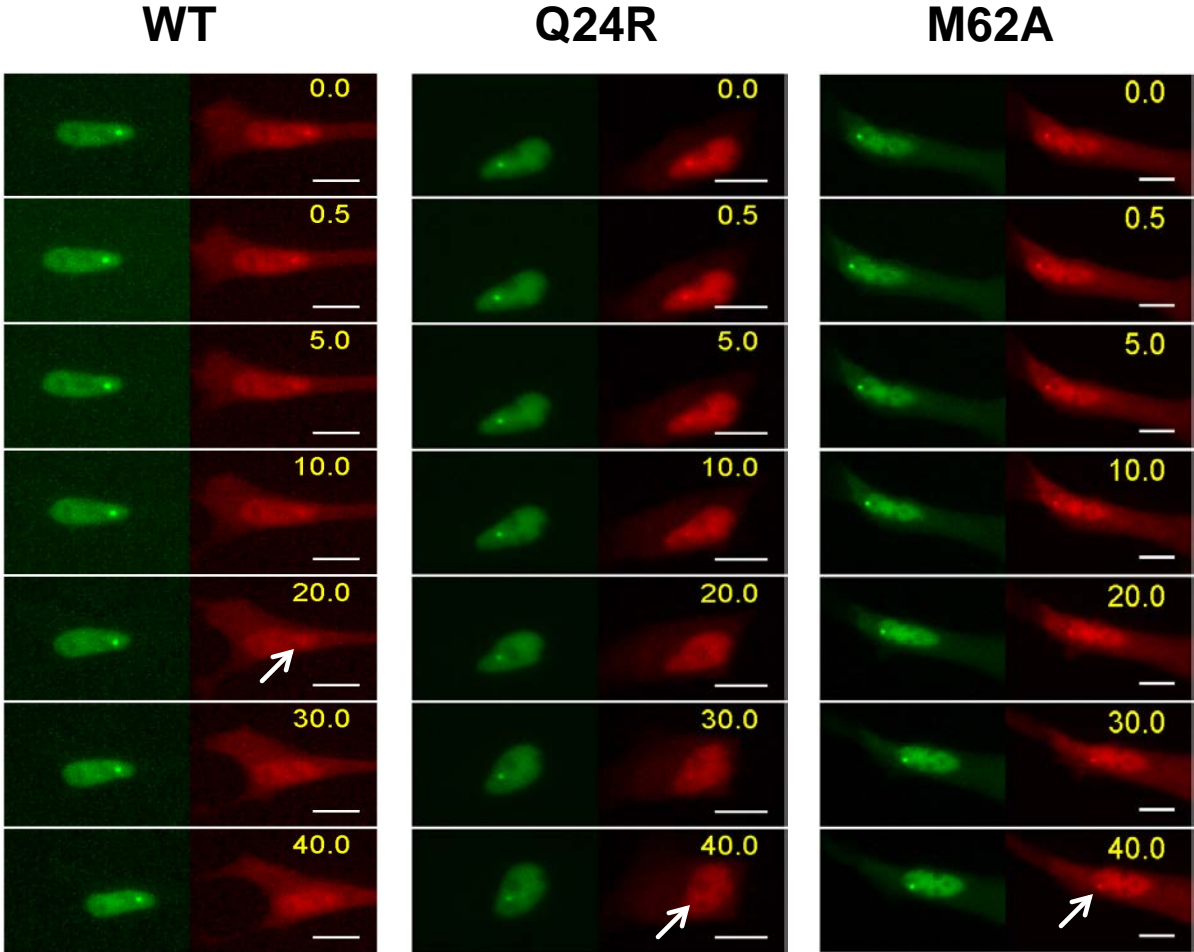

Figure S5

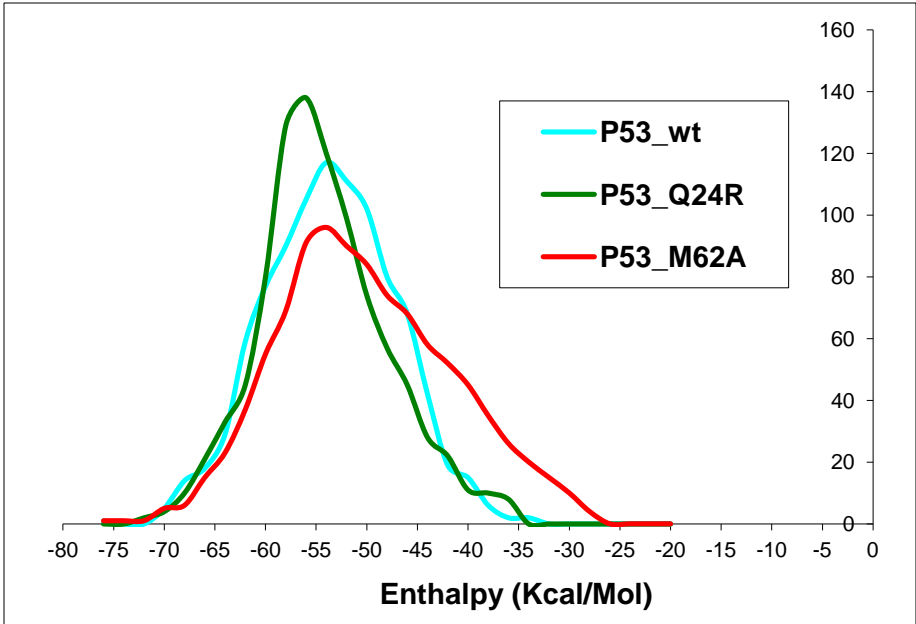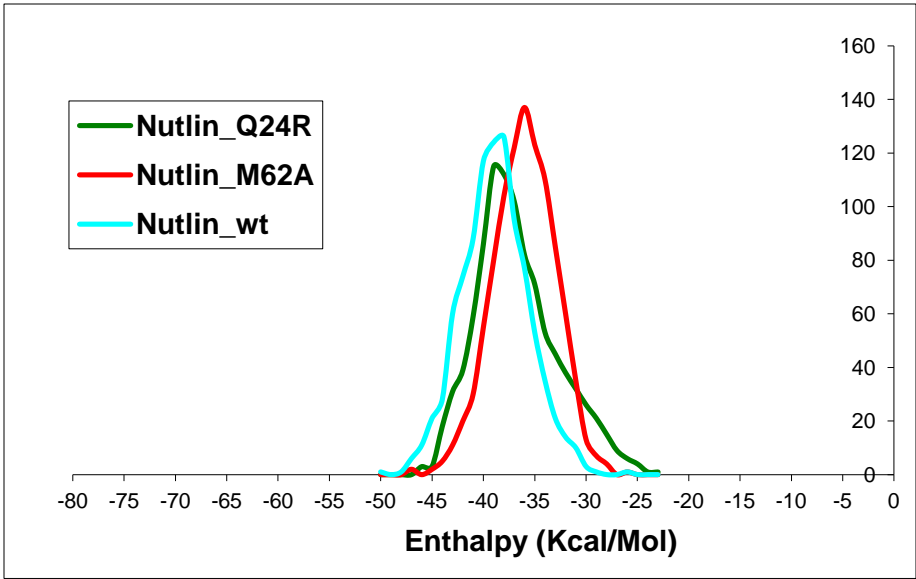

Supplement: File S1 — Supporting information Figure S1 to S6. Figure S1 Selected HDM2 variants display in vitro Nutlin-resistanc phenotype. A, in vitro pull-down assay showing reduced inhibition by Nutlin (10 µM) to binding of p53 for indicated parental HDM2 variants. Data represents binding of p53 (determined by densitometric analysis after Western blotting of 2 independent experiments) to each variant expressed as a percentage of that observed in the absence of Nutlin treatment. Values represent mean ± SD. B, as in A except for indicated point mutants. C, in vitro pull-down assay showing binding of HDM2 and variants to p53 in absence of nutlin. Binding of mutants (determined by densitometric analysis after Western blotting of 2 independent experiments) is expressed relative to binding of wild-type HDM2 to p53 (set to 100%). Values represent mean ± SD. Figure S2 Effect of Nutlin on p53-binding for HDM2 variants carrying single mutations derived from parental clone HDM2-5.3. In the presence of Nutlin (10 µM), no significant increase in p53 binding is observed for mutant HDM2 compared to wild-type. Figure S3 Nutlin shows reduced inhibition of selected HDM2 variants in p53/HDM2-null DKO cells. DKO cells co-transfected with p53 and indicated HDM2 variants. p53 function measured by reporter gene activity in presence of indicated amounts of Nutlin. Activity expressed as percentage of reporter gene transactivation seen with wild-type HDM2 (set to 100, indicated by dotted line). Values represent mean ± SD from two to three independent experiments, *p<0.5, **p<0.05, ***p<0.005. Figure S4 Time lapse images indicate persistence of mutant HDM2-p53 complex in presence of Nutlin using F2H assay. Green dot shows p53 bound to DNA. Co-localised red dot shows HDM2 in complex with p53. Arrows indicate last time point where HDM2 (wild-type or indicated mutant) is present in complex. Time is indicated in minutes. Figure S5 The distribution of energies of interactions (enthalpies) of p53 (top) and Nutlin (bo [file pone.0062564.s001.pdf]

## Slide 1
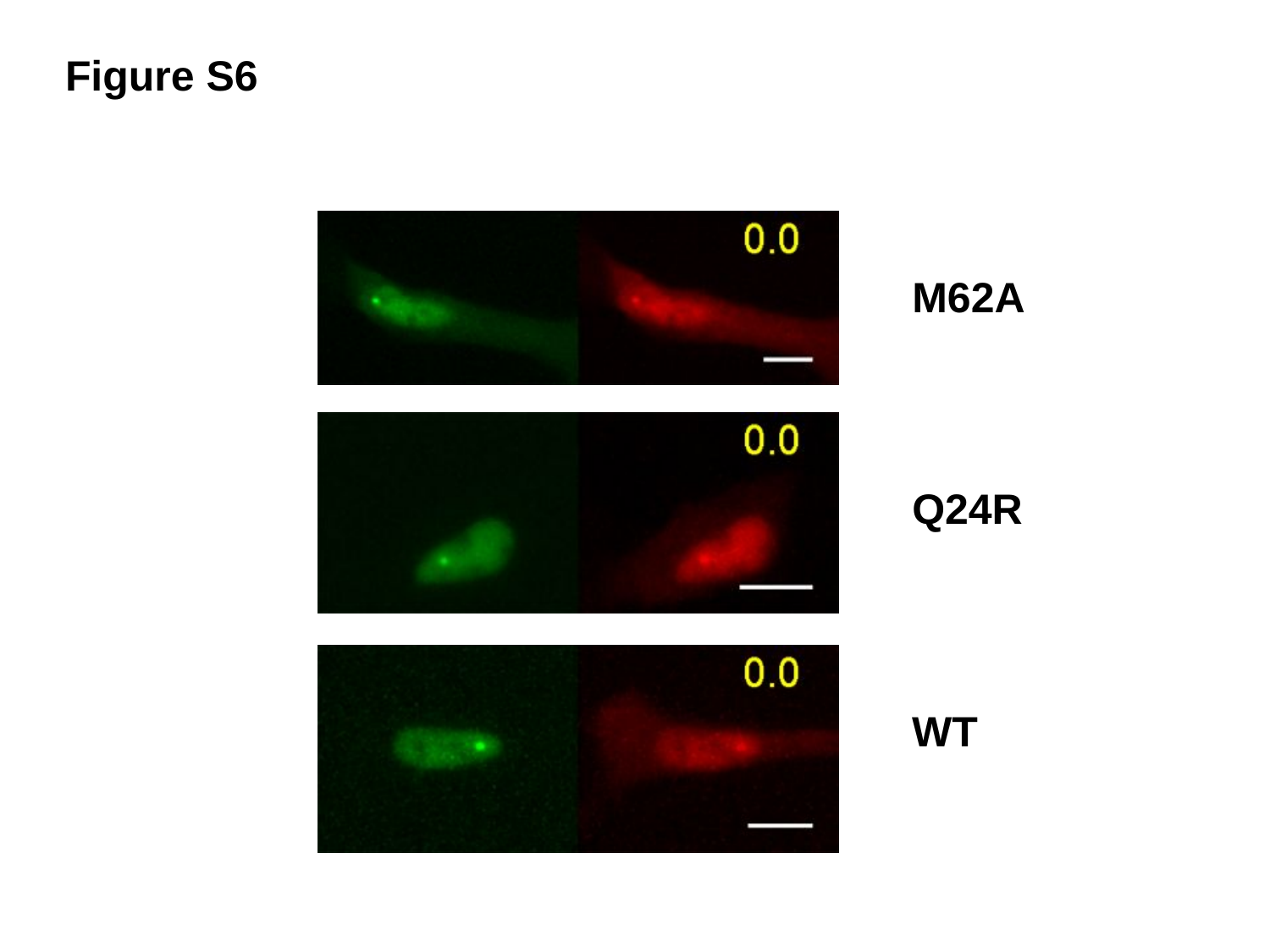

Figure S6
M62A
Q24R
WT

Supplement: Video S1 — Video footage of F2H assay measuring interaction between HDM2 (wild-type and indicated mutants) and p53 indicates persistence of mutant HDM2 interactions with p53 compared to wild-type. Green dot shows p53 bound to DNA. Co-localised red dot shows HDM2 in complex with p53. Time is indicated in minutes. (PPTX) [file pone.0062564.s002.pptx]
